# Gasotransmitter CO Attenuates Bleomycin-Induced Fibroblast Senescence via Induction of Stress Granule Formation

**DOI:** 10.1155/2021/9926284

**Published:** 2021-06-29

**Authors:** Yingqing Chen, Feng Jiang, Guangyao Kong, Shuo Yuan, Yuying Cao, Qinggao Zhang, Qianqian Wang, Liping Liu

**Affiliations:** ^1^Chronic Disease Research Center, Medical College, Dalian University, Dalian, 116622 Liaoning, China; ^2^Key Laboratory of Natural Medicines of the Changbai Mountain, Ministry of Education, College of Pharmacy, Yanbian University, Yanji, Jilin Province 133002, China

## Abstract

Cellular senescence is recognized as a phenomenon wherein a proliferative cell undergoes a permanent growth arrest. The accumulation of senescent cells over time can become harmful and result in diseases and physiological decline. Plasminogen activator inhibitor (PAI-1) is considered as a critical marker and mediator of cellular senescence. The formation of stress granules (SGs) could prevent senescence through the sequestration of PAI-1, and we previously suggested that exogenous carbon monoxide (CO) could induce SG assembly via integrated stress response (ISR). Although CO is known to possess anti-inflammatory, antioxidative, and antiapoptotic properties, whether it exerts antisenescent effect is still not well defined. Here, to address whether CO-induced SGs could protect against cellular senescence, we first treated lung fibroblasts with bleomycin (BLM) to establish DNA damage-induced cellular senescence, and observed a significant increase of several hallmarks of senescence through SA-*β*-gal staining, immunofluorescence, qRT-PCR, and Western blot assay. However, pre- and posttreatment of CO could remarkably attenuate these senescent phenotypes. According to our immunofluorescence results, CO-induced SGs could inhibit BLM-induced cellular senescence via sequestration of PAI-1, while it was abolished after the cotreatment of ISR inhibitor (ISRIB) due to the inhibition of SG assembly. Overall, our results proposed a novel role of CO in suppressing bleomycin-induced lung fibroblast senescence through the assembly of SGs.

## 1. Introduction

Cellular senescence is a process of permanent cell cycle arrest in response to various physiological and environmental stresses, including radiation (ionizing and UV), multiple anticancer drugs (bleomycin and etoposide), and oxidative stress [1]. Several studies have reported that the accumulation of senescent cells in tissues has both positive and negative consequences. The positive effects of senescent cells include tumor suppression, muscle regeneration, and skin wound healing in young organisms. Conversely, the detrimental effects of senescent cells have been observed in the context of age-related conditions, including cancer, cardiovascular diseases, neurodegeneration, diabetes, sarcopenia, and declining immune function in the elderly [2–4]. Senescent cells typically appear flattened and enlarged and show increased cytoplasmic granularity [1]. In addition, senescent cells also display several other differences from proliferative cells. These differences include the increase of senescence-associated *β*-galactosidase (SA-*β*-gal) activity, phosphorylated H2A histone family member X (*γ*-H2AX) foci, cyclin-dependent kinase inhibitors (CDKIs), such as p21^CIP1^ and p16^INK4a^, and senescence-associated secretory phenotype (SASP) which consists of growth hormones, proinflammatory cytokines, chemokines, angiogenic factors, and extracellular matrix remodeling proteases [5, 6]. Recent observations have supported that the increased secretion of serine protease inhibitor plasminogen activator inhibitor 1 (PAI-1), a component of SASP, can accelerate the aging in mice, and which is not only a marker but also a critical mediator of cellular senescence [7]. Furthermore, senescence-inducing signals such as DNA damage response (DDR) and oxidative stress usually enhance the activation of tumor suppressor p53, and which trigger the expression of PAI-1 to interfere cyclin D1-dependent phosphorylation of Rb resulting in the irreversible cell cycle arrest [8].

Due to the role of cellular senescence in diseases such as neurodegeneration, cancer, and aging-related fibrosis, it has been reported to be an attractive therapeutic target. There are mainly two strategies that are under current development [9]. The first approach focuses on identifying compounds that can specifically induce senescent cells to die, and which is termed as senolytics. Given that senescent cells are resistant to apoptosis, the mechanism involved in this resistance is a preferential target of senolytics. The second approach is aimed at reducing the negative effect of SASP. Rapamycin, resveratrol, and metformin have been shown to effectively reduce the expression of SASP through inhibition of the NF-*κ*B and mTOR signaling pathways [9, 10]. Besides these strategies, more recent studies have showed that hydrogen sulfide (H_2_S) and nitric oxide (NO), two kinds of gasotransmitters, can, respectively, delay nicotinamide-induced premature senescence and oxidative stress-induced epithelial cell senescence via reducing ROS production and enhancing SIRT1 activity [11, 12]. However, it is still unclear whether carbon monoxide (CO), one another gasotransmitter, can protect cells against stress-induced premature senescence.

CO is endogenously synthesized by heme oxygenase (HO), which can be induced by various circumstances (HO-1) or constitutively expressed in several organs (HO-2) [13]. Either applying a low concentration of exogenous CO or induction of the endogenous CO by HO-1 activation can exhibit cyto- and tissue-protective effects in various models of cellular and tissue injuries involving anti-inflammatory, antioxidant, and antiapoptotic effects [14]. Our numerous studies have demonstrated that long-term treatment of low-dose CO can protect various pathological conditions, including hepatic ischemia/reperfusion injury [15], acute lung injury [16], sepsis [17], Alzheimer's disease [18], and acetaminophen-induced liver injury [19]. Accumulative studies have reported that CO can generate low levels of mitochondrial reactive oxygen species (mtROS) via inhibition of cytochrome c oxidase, which in turn mediates integrated stress response (ISR) [20, 21]. Recently, we also proved that low levels of mtROS generated by CO could specifically induce the protein kinase R-like endoplasmic reticulum kinase (PERK)/eukaryotic translation initiation factor 2A (eIF2*α*) signaling pathway and upregulated sestrin 2 (SESN2) [22], fibroblast growth factor 21 (FGF21) [23], and parkin [19] expression to promote redox homeostasis and enhance cellular survival.

In response to diverse environmental stresses, including heat, hyperosmolarity, and oxidative stresses, eukaryotic cells temporarily turn off the protein synthesis to control energy expenditure for the repair of stress-induced damage [24]. One of the main mechanisms is the formation of stress granule (SG) in the cytoplasm, and the nonmembrane-bounded SGs can arrest mRNAs and several harmful proteins to protect cells from apoptosis [24, 25]. SG biogenesis is recognized as a conserved stress response and that can be initiated by the oligomerization of Ras GTPase-activating protein-binding protein 1 (G3BP1) and aggregation of RNA-binding proteins, including T cell intracytoplasmic antigen (TIA-1) and TIA1-related protein (TIAR) [26]. Accumulative evidence has depicted that the assembly of SGs can protect cells from apoptosis via minimizing energy expenditure, controlling proteostasis and ribostasis, and improving cell survival under damaging conditions [27]. Furthermore, SG formation can inhibit cellular senescence via the sequestration of PAI-1 into SGs and subsequently enhances the pathway of cyclin D1 to remove cell cycle arrest and delay the senescent state [28]. Intriguingly, our recent study has clearly suggested that exogenous CO can induce the formation of SGs through the induction of ISR, especially through the PERK-eIF2*α* signaling pathway [29]. However, whether CO-induced SGs can prevent stress-induced premature senescence is still not well defined.

In this study, to construct DNA damage-induced senescence, we treated human and mouse fibroblasts with bleomycin (BLM) that can cause chromosomal instability, and we found that the administration of CO-releasing molecular (CORM-A1) and exogenous CO gas could effectively inhibit the cellular senescence, and the induction of SGs could sequestrate PAI-1 to recover the cyclin D1 signaling pathway to ameliorate cell cycle arrest. Moreover, we showed that ISR inhibitor (ISRIB) could abolish the protective effect of CO on BLM-induced premature senescence via suppressing the formation of SGs and enhancing the secretion of PAI-1. Our data indicated a novel mechanism whereby CO could protect against DNA damage-mediated cellular senescence through the induction of SG assembly and sequestration of PAI-1, in the cytoplasm, to delay the process of cellular senescence.

## 2. Materials and Methods

### 2.1. Reagents and Chemicals

CO-releasing molecular-A1 (CORM-A1), thapsigargin (Tg), PAI-1 inhibitor (TM5441), and ISR inhibitor (ISRIB) were purchased from Sigma-Aldrich (St. Louis, MO, USA). Bleomycin (BLM) was purchased from MedChemExpress LLC (MCE, Monmouth Junction, NJ).

### 2.2. Cell Culture

Human Caucasian fibroblast-like fetal lung cells (WI-38 cells) were cultured in Minimum Essential Medium (MEM, Thermo Fisher Scientific, Waltham, MA, USA) with 10% fetal bovine serum (FBS, BI, USA) and 1% penicillin-streptomycin (Gibco, USA) solution at 37°C in a humidified incubator with 5% CO_2_. We isolated primary mouse embryonic fibroblasts (MEFs) from 8-week female pregnant C57BL/6 mice and cultured in DMEM (Gibco, Grand Island, NY) medium with 10% fetal bovine serum, 1% penicillin-streptomycin solution, and 1% MEM nonessential amino acid solution (N1250, Solarbio, Beijing, China).

### 2.3. Transfection of siRNAs

Small interfering RNA (siRNA) against human PA I-1 (siPAI-1, sc-36179) and negative control siRNA (scRNA, sc-37007) were purchased from Santa Cruz Biotechnology, Inc. (Santa Cruz, CA, USA). WI-38 cells were seeded in a 6-well plate at a concentration of 3 × 10^5^ cells per well, and cells were transfected with siPAI-1 and scRNA, respectively, by utilizing Lipofectamine 2000 (Invitrogen, Carlsbad, USA) in accordance with the manufacturer's protocol. In brief, dilute 100 pmol siRNA oligomer in 250 ml Opti-MEM (Gibco, USA) without serum and mix gently. Dilute 5 ml Lipofectamine 2000 in 250 ml Opti-MEM without serum and mix gently for 5 min at room temperature. After a 5-minute incubation, combine the diluted oligomer with the diluted Lipofectamine 2000. Mix gently and incubate for 20 min at room temperature. Drop the 500 ml oligomer-Lipofectamine 2000 complexes to each well containing cells and medium. Mix gently by rocking the plate back and forth. After a 36-hour transfection, cells were additionally treated with BLM for 96 h. Then, the cells were collected for SA-*β*-gal staining, qRT-PCR, and Western blot assay.

### 2.4. RNA Isolation and Quantitative Real-Time Polymerase Chain Reaction (qRT-PCR)

Total RNA was isolated from WI-38 cells and MEFs by utilizing TRIzol reagent (Takara, Otsu, Japan), according to the manufacturer's instructions. In brief, 2 *μ*g of total RNA was used to generate cDNA using Prime Script™ RT Reagent kit with gDNA Eraser (Takara, Otsu, Japan). The synthesized cDNA was subjected to PCR-based amplification. To perform quantitative real-time PCR (qRT-PCR), the synthesized cDNA was amplified with TB Green Premix EX Taq™ II (Takara, Otsu, Japan) on Bio-Rad CFX Connect™ Real-Time PCR Detection System (Bio-Rad Laboratories). The following primers were human GAPDH (forward 5′-CAA TGA CCC CTT CAT TGA CCT C-3′, reverse 5′-AGC ATC GCC CCA CTT GAT T-3′), human p21 (forward 5′-CGA TGG AAC TTC GAC TTT GTC A-3′, reverse 5′-GCA CAA GGG TAC AAG ACA GTG-3′), human IL-6 (forward 5′-ACT CAC CTC TTC AGA ACG AAT TG-3′, reverse 5′-CCA TCT TTG GAA GGT TCA GGT TG-3′), human TNF-*α* (forward 5′-GCT GCA CTT TGG AGT GAT CG-3′, reverse 5′-GTT TGC TAC AAC ATG GGC TAC AG-3′), human IL-1*β* (forward 5′-TTA CAG TGG CAA TGA GGA TGA C-3′, reverse 5′-GTC GGA GAT TCG TAG CTG GAT-3′), human PAI-1 (forward 5′-TGA TGG CTC AGA CCA ACA AAG-3′, reverse 5′-CAG CAA TGA ACA TGC TGA GG-3′), mouse GAPDH (forward 5′-GGG AAG CCC ATC ACC ATC T-3′, reverse 5′-CGG CCT CAC CCC ATT TG-3′), mouse p21 (forward 5′-GTG GCC TTG TCG CTG TCT T-3′, reverse 5′-GCG CTT GGA GTG ATA GAA ATC TG-3′), mouse IL-6 (forward 5′-CCA GAG ATA CAA AGA AAT GAT GG-4′, reverse 5′-ACT CCA GAA GAC CAG AGG AAA T-3′), mouse TNF-*α* (forward 5′-AGA CCC TCA CAC TCA GAT CAT CTT C-3′, reverse 5′-TTG CTA CGA CGT GGG CTA CA-3′), and mouse IL-1*β* (forward 5′-TCG CTC AGG GTC ACA AGA AA-3′, reverse 5′-ATC AGA GGC AAG GAG GAA ACA C-3′).

### 2.5. Western Blot

Cell lysates were prepared using protein extraction kit (Solarbio, Beijing, China) containing protease inhibitor and phosphatase inhibitors. Total protein concentration of the lysates was measured using a BCA protein assay kit (Solarbio, Beijing, China). Proteins were resolved by SDS-PAGE, transferred onto polyvinylidene difluoride (PVDF) membrane (Millipore, Darmstadt, Germany), and probed with appropriate dilutions of the following antibodies: cyclin D1 (1 : 1000, 2922S, Cell Signaling), Lamin A/C (1 : 1000, sc-6215, Santa Cruz), TERT (1 : 500, abs136649, Absin), and *β*-actin (1 : 1000, 4967S, Cell Signaling). Then, the membranes were incubated with secondary antibody in the room temperature for 40 min. Antibody binding was visualized with an ECL chemiluminescence system (GE Healthcare Bio-Sciences, Little Chalfont, UK), and chemiluminescence signal was read by Bio-Rad ChemiDoc XRS+ (Bio-Rad Laboratories, Hercules, CA). The relative band density was analyzed by using ImageJ2x software (US National Institutes of Health, Bethesda, USA).

### 2.6. Nuclear and Cytosolic Fractions

To check the nuclear translocation of cyclin D1, the nuclear and cytoplasmic proteins of cultured WI-38 cells were separated by Nuclear/Cytosol Fraction Kit (BioVision, USA) according to the manufacturer's protocol. In brief, harvested cells were added 100 *μ*l of cytosol extraction buffer (CEB) contacting DTT and protease inhibitors. After vortex and 10 min incubation on ice, the extracts were centrifuged at 16,000 *g* for 5 min at 4°C and the supernatant was immediately removed to separate the cytoplasmic fraction from the nuclei. The nuclei pallets were then added 50 *μ*l nuclei extraction buffer (NEB), vortexed briefly, and set on ice every 10 min for a total 40 min. After centrifuging at 16,000 *g* for 10 min at 4°C, the supernatant nuclear extracts were stored at -80°C for future use.

### 2.7. SA-*β*-gal Staining

To observe the senescent cells, WI-38 and MEF cells were treated with bleomycin to construct DNA damage-induced premature cellular senescence. Then, senescence-associated- (SA-) *β*-galactosidase (gal) staining was performed by utilizing a cellular senescence cell histochemical stain kit (Sigma, CS0030) according to the manufacturer's protocol. For lung tissue staining, the left part of lung tissues was fixed at room temperature for 2 h, with a solution containing 2% formaldehyde and 0.2% glutaraldehyde in PBS, and washed three times with PBS. Then, lung tissues were incubated overnight at 37°C (in the absence of CO_2_) with the staining solution containing X-gal in N,N-dimethylformamide (pH 6.0).

### 2.8. Immunofluorescence

To observe the formation of SGs and the sequestration of PAI-1 and the *γ*-H2AX foci, WI-38 cells were plated on a 4-well Lab-Tek chambered cover glass (Nunc, Thermo Scientific, Waltham, MA) and pretreated with CORM-A1 (40 *μ*M) for 6 h followed by the administration of 25 *μ*g/ml bleomycin for 96 h to construct DNA damage-induced premature cellular senescence. Then, cells were washed in 1× PBS, fixed with 4% (*v*/*v*) paraformaldehyde in PBS at room temperature for 15 min, and permeabilized with 0.1% (*v*/*v*) Triton X-100 in PBS for 5 min. Then, cells were washed three times with PBS before incubation for 2 h at room temperature with primary antibodies (anti-TIA1, 1 : 500, ab140595, Abcam; anti-G3BP1, 1 : 200, sc-365338, Santa Cruz; anti-PAI-1, 1 : 200, sc-5297, Santa Cruz; and anti-*γ*-H2AX, 1 : 200, ab81299, Abcam) diluted in 1% (*w*/*v*) BSA in PBS. Cells were washed further three times with 1× PBS before incubation for 1 h with secondary fluorophore-coupled antibodies (Alexa Fluor 594 goat anti-rabbit IgG, 1 : 500, Invitrogen and Alexa Fluor 488 rabbit anti-mouse IgG, 1 : 400, Invitrogen), respectively. Secondary antibodies were diluted in 1% (*w*/*v*) BSA in 1× PBS. After incubation, cells were washed three times with 1× PBS and were imaged with an Olympus FV1000 confocal microscope (Olympus, Tokyo, Japan). Quantifications from immunofluorescence images were done by counting cells with TIA1 and G3BP1 colocalized SGs per total number of cells in randomly selected field. Each field contained at least 30 cells, and three images per condition were analyzed.

### 2.9. Measurement of PAI-1 and Several SASP Secretions

WI-38 cells were pretreated with 40 *μ*M CORM-A1 for 6 h followed by the administration of 25 *μ*g/ml for 96 h. During the process of cellular senescence, WI-38 cells were posttreated with 40 *μ*M CORM-A1 for 6 h every two days. After a 4-day incubation, cultured supernatant was collected, and the concentrations of PAI-1 and several SASPs, including IL-6, TNF-*α*, and IL-1*β*, were measured by human PAI-1 (DuoSet ELISA DY9387-05, BD Biosciences), human IL-6 (Cusabio Biotech, Wuhan, China), human TNF-*α* (Cusabio Biotech, Wuhan, China), and human IL-1*β* (Cusabio Biotech, Wuhan, China) ELISA kit according to the manufacturer's instructions.

### 2.10. Statistics

For statistical comparison, all values were expressed as the mean ± SD. Statistical differences between all experimental groups were applied by one-way ANOVA with Tukey's post hoc test, and all statistical analyses were assessed by GraphPad Prism software version 5.03 (San Diego, CA). The statistically significant changes among groups were considered as probability values of *p* ≤ 0.05.

## 3. Results

### 3.1. CO Inhibits Bleomycin-Induced Cellular Senescence in Human and Mouse Fibroblasts

**Figure 1:**
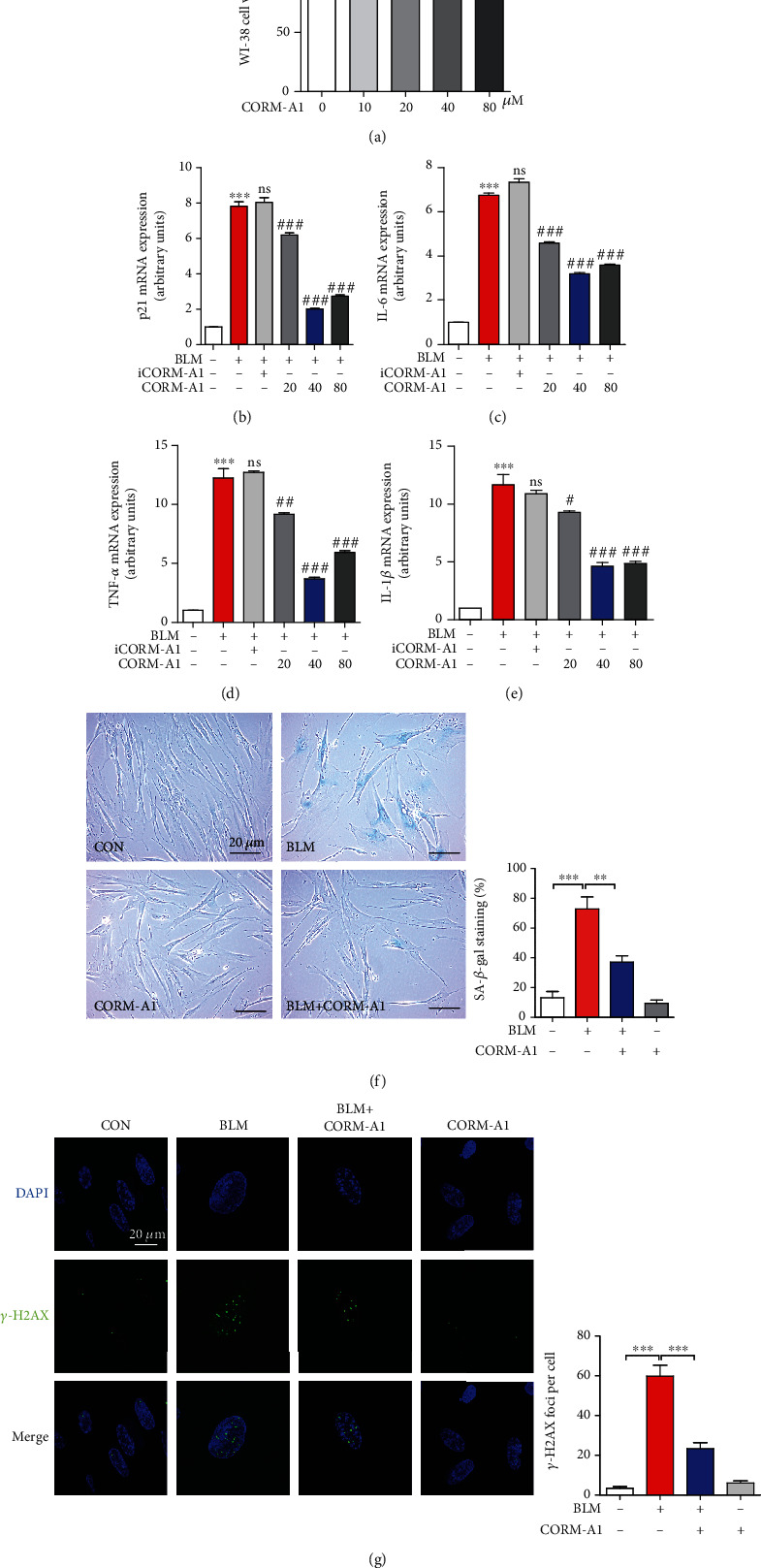
CORM-A1 inhibits BLM-induced cellular senescence in human diploid lung fibroblasts. (a) WI-38 cells were treated with various concentrations of CORM-A1 (0, 10, 20, 40, and 80 *μ*M) for 6 h, and MTT assay was performed to assess the cell viability. (b–e) WI-38 cells were pretreated with CORM-A1 (0, 20, 40, and 80 *μ*M) and iCORM-A1 (40 *μ*M) for 6 h followed by the challenge of bleomycin (BLM, 25 *μ*g/ml) for 96 h. During the process of senescence, cells were posttreated with CORM-A1 (0, 20, 40, and 80 *μ*M) for 6 h every other day. After a 4-day incubation, the mRNA expressions of p21 (b), IL-6 (c), TNF-*α* (d), and IL-1*β* (e) were measured by quantitative real-time- (qRT-) PCR. Quantitative data are expressed as the means ± SD; *n* = 3. ^∗∗∗^*p* < 0.001 vs. the vesicle control group. ^#^*p* < 0.05, ^##^*p* < 0.01, and ^###^*p* < 0.001 vs. the BLM-alone treatment group. Then, senescence-associated- (SA-) *β*-gal staining (f) and immunofluorescence for detecting *γ*-H2AX foci (g) were applied in the pre- and posttreatment of CORM-A1 (40 *μ*M). Quantitative data are expressed as the means ± SD (*n* = 3 determined in three independent experiments). ^∗∗^*p* < 0.01 and ^∗∗∗^*p* < 0.001. ns: not significant.

Bleomycin, a widely used antineoplastic drug, is well known for its side effects on induction of severe interstitial pulmonary fibrosis (IPF). Due to its combination with iron that reduces molecular oxygen to superoxide and hydroxyl radicals, bleomycin can induce DNA injury and further lead to single- and double-stranded breaks [30]. Increasing studies have demonstrated that bleomycin-mediated pulmonary fibrosis is mainly caused by lung epithelial and fibroblast senescence via activating DNA damage response (DDR) [30, 31]. To identify whether exogenous CO exerted inhibitory effect on bleomycin-induced fibroblast senescence, we first checked the cellular toxicity of CO-releasing molecular A1 (CORM-A1) on human fibroblast-like fetal lung cells, WI-38 cells, with various concentrations (0, 10, 20, 40, and 80 *μ*M) for 6 h. As we expected, low-dose CORM-A1 showed no toxicity on WI-38 cells (Figure 1(a)). Then, cells were pretreated with CORM-A1 in a dose-dependent manner (0, 20, 40, and 80 *μ*M) for 6 h followed by the administration of bleomycin (25 *μ*g/ml) for 96 h. During the challenge of bleomycin, cells were posttreated with CORM-A1 for 6 h every two days. After a 4-day incubation, we found that bleomycin alone significantly increased the number of cells positive for the expression of p21 (Figure 1(b)) and several SASPs, including IL-6, TNF-*α*, and IL-1*β* (Figures 1(c)–1(e)). However, pre- and posttreatment of CORM-A1 could significantly reduce the levels of p21 and SASPs stimulated by bleomycin, indicating that CORM-A1 might possess antisenescent effect on bleomycin-induced premature senescence. As a negative control, herein, we treated cells with inactive CORM-A1 (iCORM-A1) that is incapable of releasing CO and found that iCORM-A1 showed no interference in p21 and SASP levels in the presence of bleomycin (Figures 1(b)–1(e)). Given that 40 *μ*M CORM-A1 showed optimally to reduce the levels of p21 and SASP, in the following studies, we chose 40 *μ*M as the appropriate dose to treat cells.

**Figure 2:**
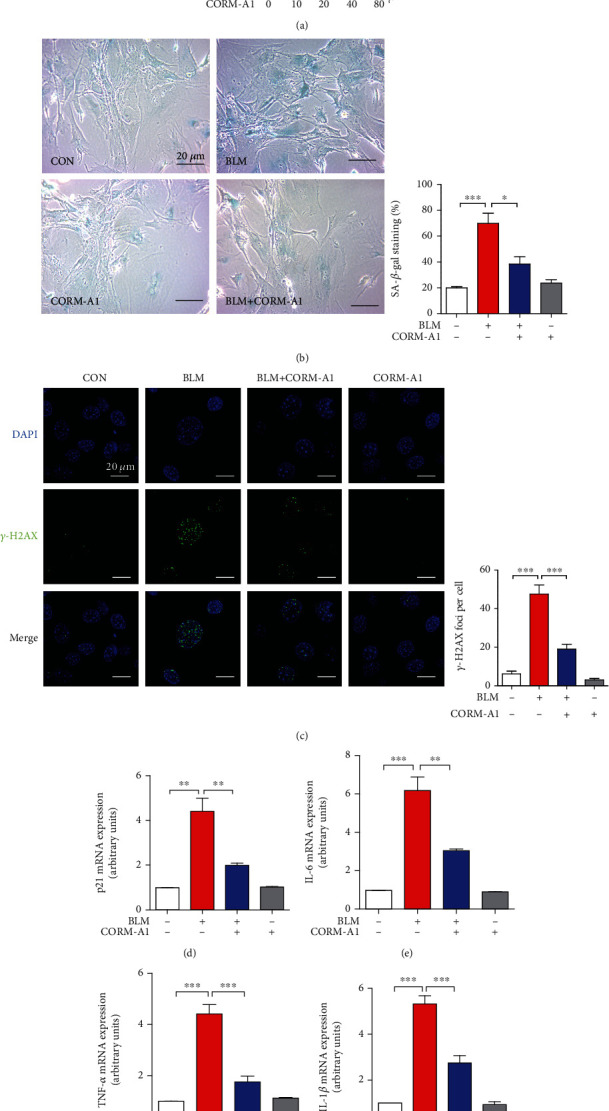
CORM-A1 attenuates BLM-induced cellular senescence in mouse embryonic fibroblasts. (a) Mouse embryonic fibroblasts (MEFs) were treated with CORM-A1 in a dose-dependent manner (0, 10, 20, 40, and 80 *μ*M) for 6 h, and MTT assay was performed to assess the cell viability. MEFs were pretreated with CORM-A1 (40 *μ*M) for 6 h prior to the stimulation of BLM (25 *μ*g/ml) for 48 h. Then, SA-*β*-gal staining (b) and *γ*-H2AX foci (c) were detected, and the mRNA expressions of p21 (d), IL-6 (e), TNF-*α* (f), and IL-1*β* (g) were detected by qRT-PCR. Quantitative data are expressed as the means ± SD (*n* = 3 determined in three independent experiments). ^∗^*p* < 0.05, ^∗∗^*p* < 0.01, and ^∗∗∗^*p* < 0.001. ns: not significant.

To further assess the antisenescent effect of CORM-A1, we treated WI-38 cells (Figure 1) and primary mouse embryonic fibroblasts (MEFs) (Figure 2) with CORM-A1 in the stimulation of bleomycin and found that the enhanced markers of cellular senescence, including the percentage of senescence-associated (SA)-*β*-gal-positive cells (Figures 1(f) and 2(b)), *γ*-H2AX foci (Figures 1(g) and 2(c)), mRNA expression of p21 (Figure 2(d)), IL-6 (Figure 2(e)), TNF-*α* (Figure 2(f)), and IL-1*β* (Figure 2(g)), were all significantly decreased. Due to p21 is a well-known target gene of p53, and which plays a critical role in the process of cellular senescence [1, 5, 6], we next checked the protein levels of p53 and p21 in WI-38 cells. Consistent with the results obtained from the regulation of senescent transcripts, the pretreatment of CORM-A1 could remarkably decrease the protein levels of p53 and p21 in the challenge of bleomycin (Figures S2A–S2C). To further investigate whether exogenous CO gas could also protect against cellular senescence, we next treated WI-38 cells with 250 ppm CO gas for 6 h every two days in the presence or absence of bleomycin (25 *μg/ml*) for 96 h. Our results revealed that the exogenous CO gas could remarkably inhibit the corresponding senescence markers induced by bleomycin (Figure S1). According to these results, we clearly demonstrated that low-dose administration of CORM-A1 and CO gas could effectively prevent bleomycin-induced premature senescence.

### 3.2. PAI-1 Is Involved in Bleomycin-Induced Premature Senescence in WI-38 Cells

**Figure 3:**
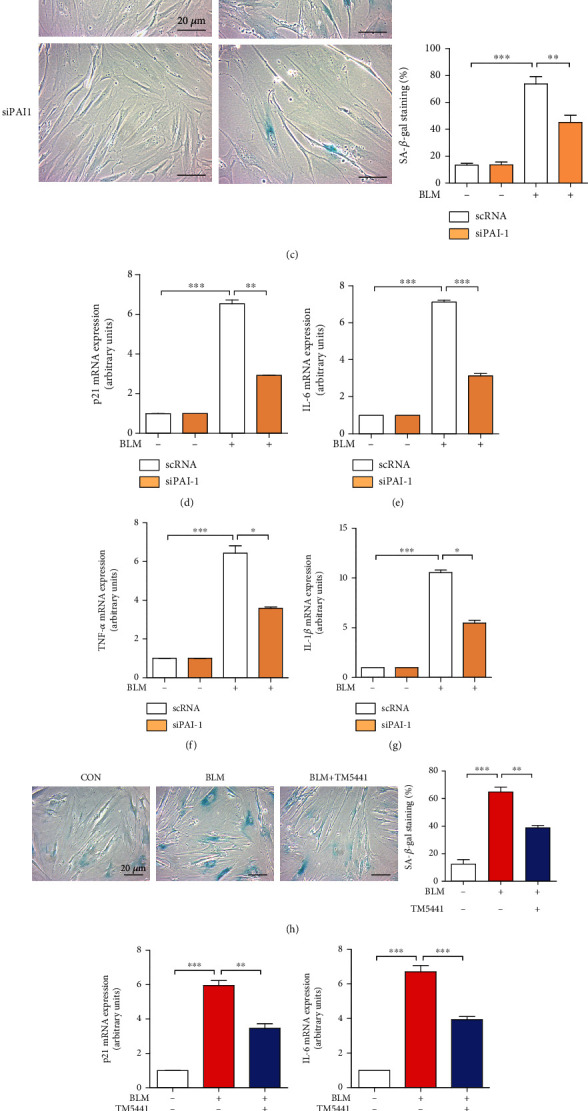
PAI-1 is involved in BLM-induced fibroblast senescence. (a) WI-38 cells were treated with BLM (25 *μ*g/ml) for 96 h, and the mRNA expression of PAI-1 was detected by qRT-PCR. (b) WI-38 cells were transfected with scramble siRNA (scRNA) and siRNA against PAI-1 (siPAI-1) for 36 h, and then, PAI-1 mRNA level was assessed by qRT-PCR. Transfected cells were treated with BLM (25 *μ*g/ml) for 96 h, and cells were performed SA-*β*-gal staining (c), and the mRNA expressions of p21 (d), IL-6 (e), TNF-*α* (f), and IL-1*β* (g) were measured by qRT-PCR. WI-38 cells were cotreated with PAI-1 inhibitor, TM5441 (10 *μ*M), and BLM (25 *μ*g/ml) for 96 h. Then, SA-*β*-gal staining (h) and the mRNA expressions of p21 (i), IL-6 (j), TNF-*α* (k), and IL-1*β* (l) were measured by qRT-PCR. Quantitative data are expressed as the means ± SD (*n* = 3 determined in three independent experiments). ^∗^*p* < 0.05, ^∗∗^*p* < 0.01, and ^∗∗∗^*p* < 0.001.

Plasminogen activator inhibitor 1 (PAI-1) is a primary inhibitor of tissue-type and urokinase-type plasminogen activators, which can inhibit the conversion of plasminogen into plasmin, and plays a major role in fibrogenesis [32]. Accumulating evidences have suggested that PAI-1 expression is significantly increased in senescent cells, and which might be not only a marker but also a key mediator of cellular senescence and organismal aging [7]. To determine whether PAI-1 was essential for bleomycin-incubated cellular senescence, we first checked the mRNA expression of PAI-1 in WI-38 cells. Our results showed that bleomycin could induce a significant enhancement of PAI-1 (Figures 3(a) and S2D). Next, to evaluate whether increased PAI-1 expression was involved in the bleomycin-induced premature senescence, we transfected cells with siRNA against PAI-1 (Figures 3(b) and S2D), and then cells were stimulated with bleomycin. We found that, in the administration of bleomycin, silencing PAI-1 could dramatically decreased the markers of senescence, including the percentage of SA-*β*-gal stained cells (Figure 3(c)) and the expression of p21 (Figures 3(d) and S2D), p53 (Figure S2D), IL-6 (Figure 3(e)), TNF-*α* (Figure 3(f)), and IL-1*β* (Figure 3(g)) compared with cells transfected with scramble RNA (scRNA). Furthermore, PAI-1 inhibitor, one small molecule TM5441 [33], also exhibited a significant inhibitory effect on bleomycin-induced cellular senescence. Our data showed that the treatment of TM5441 (10 *μ*M) in the presence of bleomycin (25 *μg/ml*) could significantly decrease SA-*β*-gal-positive cells (Figure 3(h)) and mRNA expression of several SASPs (Figures 3(i)–3(l)). These data strongly demonstrated that PAI-1 played a critical role in bleomycin-induced premature senescence, and the inhibition of PAI-1 could effectively ameliorate the process of senescence.

### 3.3. CO-Induced SGs Participate in Reducing Bleomycin-Induced Senescence by PAI-1 Sequestration

**Figure 4:**
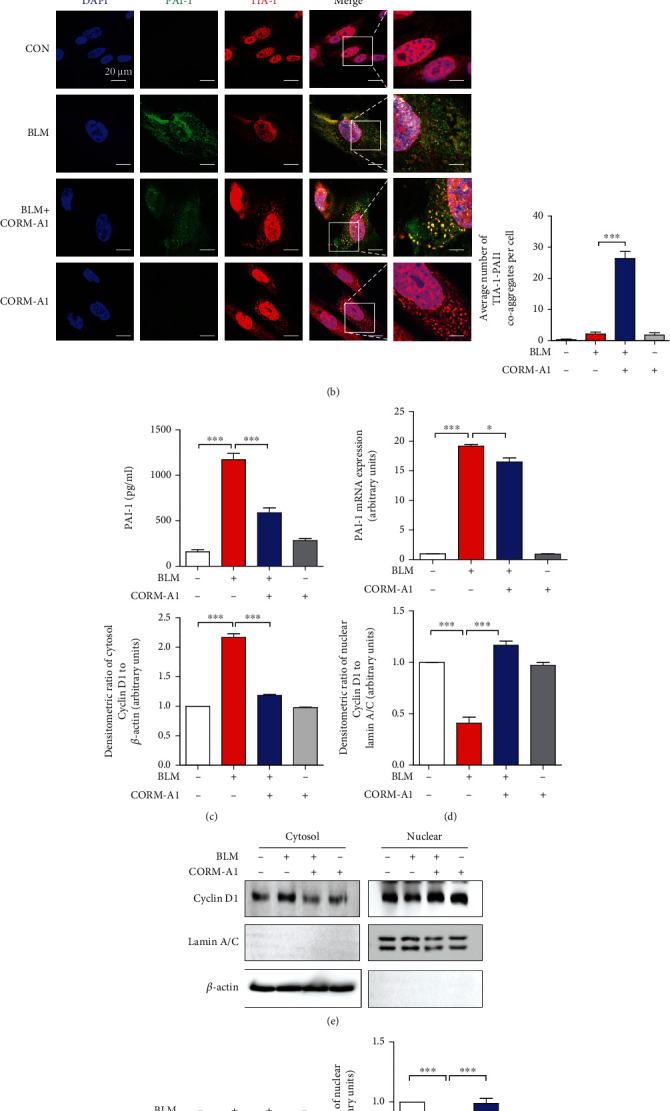
CO inhibits secretion of PAI-1 in senescent cells via SG formation. (a) WI-38 cells were treated with CORM-A1 in various concentrations (0, 20, 40, and 80 *μ*M) and iCORM-A1 (40 *μ*M) for 6 h. As a positive control, WI-38 cells were treated with 200 nM thapsigargin (Tg) for 45 min, and then, immunofluorescence assay was performed to detect the formation of SGs by visualizing the colocalization of TIA-1(red) and G3BP1 (green). (b) WI-38 cells were pretreated with CORM-A1 (40 *μ*M) for 6 h prior to the administration of bleomycin (25 *μ*g/ml) for 96 h. After a 4-day treatment, immunofluorescence assay was performed to detect the TIA-1 (red) and PAI-1 (green) coaggregates. The secretion of PAI-1 was assessed by ELISA (c), and the mRNA expression of PAI-1 was assessed by qRT-PCR (d). (e) Nuclear translocation of cyclin D1 was measured by Western blot assay in cytosol and nuclear fractions. (f) The phosphorylated and total forms of Rb were analyzed by Western blot assay. The secretion of SASP, including IL-6 (g), TNF-*α* (h), and IL-1*β* (i), was measured by ELISA. Quantitative data are expressed as the means ± SD (*n* = 3 determined in three independent experiments). ^∗∗^*p* < 0.01 and ^∗∗∗^*p* < 0.001. ns: not significant.

One previous study has demonstrated that the assembly of SGs induced by continuous mild oxidative stress can decrease the number of senescent cells through the sequestration of PAI-1 and subsequently activates the cyclin D1-dependent signaling pathway to maintain proliferative state [28]. Our recent study has reported that low-dose exogenous CO can stimulate SG formation by selective induction of the PERK-eIF2*α* signaling pathway, one branch of integrated stress response (ISR) [29]. Based on these reports, we hypothesized that the antisenescent effect of CO might be mediated by SG formation. Here, we first confirmed the beneficial effect of CO on SG assembly by treating WI-38 cells with various concentrations of CORM-A1 (0, 20, 40, and 80 *μ*M) for 6 h. Immunofluorescence was applied to check the amount of SGs by applying anti-TIA-1 and anti-G3BP1 antibodies. According to our results, 40 *μ*M and 80 *μ*M CORM-A1 (Figure 4(a)) could significantly increase the assembly of TIA-1- and G3BP-1-positive SGs in the cytoplasm, and iCORM-A1 revealed no benefits on SG assembly (Figure 4(a)), suggesting again that CORM-A1 exerted an inducible effect on SG formation. In further study, we tried to check whether CO could stimulate the sequestration of PAI-1 into SGs in the stimulation of bleomycin. As we expected, the increased number of SGs induced by CORM-A1 could effectively sequestrate PAI-1 through detecting the coaggregation of TIA-1 and PAI-1 (Figure 4(b)). Simultaneously, we evaluated the secretion of PAI-1 by ELISA assay. Cells treated with bleomycin alone significantly increased the secretion of PAI-1, while CORM-A1 administration could dramatically attenuated the level of PAI-1 in cultured medium (Figure 4(c)). To define whether the decreased secretion of PAI-1 was associated with the transcription of PAI-1, we next assessed the mRNA level of PAI-1, and our data showed that CORM-A1 could slightly decreased the transcripts (Figure 4(d)), indicating that the secretion of PAI-1 regulated by CORM-A1 was mainly resulted from the induction of SGs rather than the downregulation of PAI-1 transcription. It has been reported that PAI-1 secretion correlates with the onset of senescence via inhibition of cyclin D1 nuclear translocation and hypophosphorylation of Rb [34]. To check the level of cyclin D1 in the nucleus, we isolated cytosol and nuclear fractions from WI-38 cells and found that the level of cyclin D1 was significantly decreased in bleomycin-treated cells, while it was significantly increased in the fraction of the cytoplasm (Figure 4(e)). In addition, CORM-A1 could positively regulate the nuclear translocation of cyclin D1 (Figure 4(e)), and which is associated with the increase of Rb phosphorylation (Figure 4(f)). Moreover, we also observed that the secretions of SASP, including IL-6 (Figure 4(g)), TNF-*α* (Figure 4(h)), and IL-1*β* (Figure 4(i)), induced by bleomycin were dramatically reduced in the administration of CORM-A1. Our findings demonstrated that CO-mediated downregulation of PAI-1 secretion was associated with the SG formation, and which could enhance the activation of cyclin D1 and hyperphosphorylation of Rb to delay the process of senescence and the secretion of SASP.

### 3.4. ISRIB Abolishes CO-Induced SG Assembly and Recovers Bleomycin-Mediated Cellular Senescence

**Figure 5:**
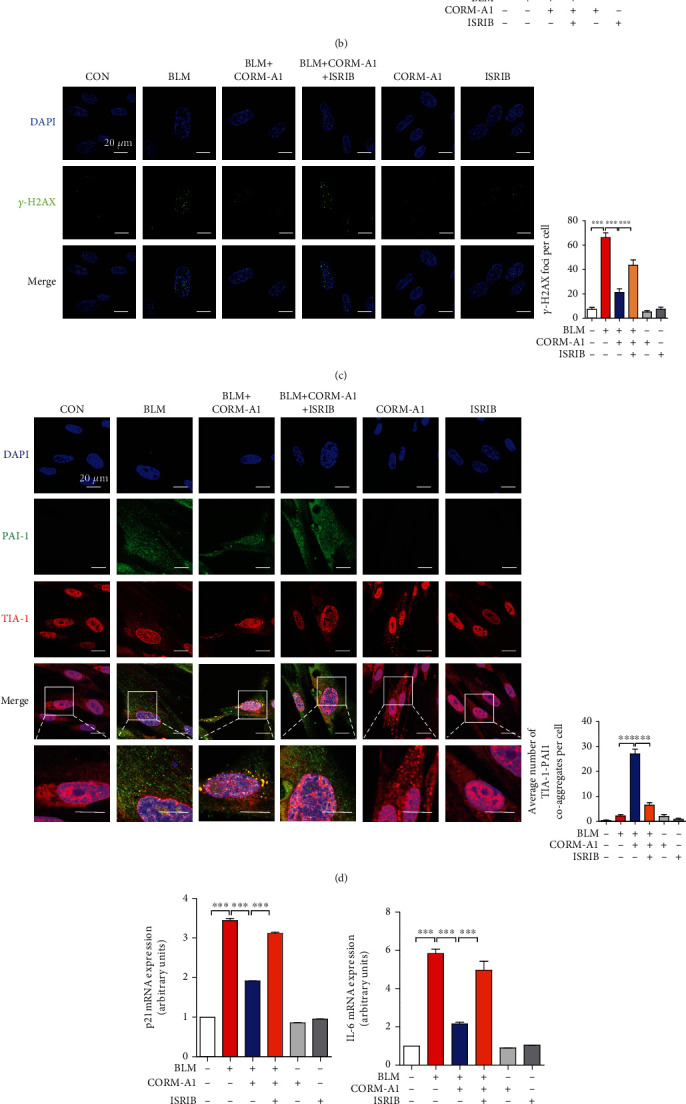
ISRIB abrogates CO-mediated SG formation and recovers BLM-induced cellular senescence. (a) WI-38 cells were treated with 40 *μ*M CORM-A1 in the presence or absence of ISRIB (200 nM) for 6 h. Immunofluorescence assay was performed to detect the formation of SGs by visualizing the colocalization of TIA-1(red) and G3BP1 (green). WI-38 cells were pretreated with CORM-A1 (40 *μ*M) with or without ISRIB (200 nM) for 6 h followed by the challenge of bleomycin (25 *μ*g/ml) for 96 h. Then, SA-*β*-gal staining (b) and *γ*-H2AX foci (c) were detected, and immunofluorescence assay (d) was performed to assess the coaggregates of TIA-1 (red) and PAI-1 (green). The mRNA expressions of p21 (e), IL-6 (f), TNF-*α* (g), and IL-1*β* (h) by qRT-PCR. The secretions of PAI-1 (i), IL-6 (j), TNF-*α* (k), and IL-1*β* (l) were detected by ELISA. Quantitative data are expressed as the means ± SD (*n* = 3 determined in three independent experiments). ^∗^*p* < 0.05, ^∗∗^*p* < 0.01, and ^∗∗∗^*p* < 0.001.

According to our recent study, CO-induced SG assembly was dependent on the ISR signaling pathway, and ISR inhibitor, ISRIB, could decrease the formation of SGs in the administration of CORM2 and CO gas [29]. To confirm these reported results, we first cotreated WI-38 cells with CORM-A1 and ISRIB for 6 h and observed that ISRIB could significantly inhibit the formation of SGs by visualizing the coaggregation of TIA-1 and G3BP1 in the cytoplasm (Figure 5(a)). Next, to analyze whether the inhibition of SGs by ISRIB could negatively regulate the antisenescent effect of CORM-A1, we next performed pre- and posttreatment of CORM-A1 in WI-38 cells with or without ISRIB for 6 h followed by the administration of bleomycin for 96 h. After a 4-day incubation, we checked the percentage of SA-*β*-gal-positive cells (Figure 5(b)) and the number of *γ*-H2AX foci per cell (Figure 5(c)). In bleomycin-induced senescent cells, the enhanced activity of SA-*β*-gal and *γ*-H2AX foci was significantly decreased by CORM-A1, and which was reversed in the cotreatment of ISRIB (Figures 5(b) and 5(c)). Moreover, we also observed that ISRIB strongly inhibits the sequestration of PAI-1 into CO-induced SGs (Figure 5(d)), and for that reason, the inhibitory effect of CORM-A1 on PAI-1 secretion was drastically abolished (Figure 5(i)). Next, we also checked the mRNA expression of p21 (Figure 5(e)), several SASPs (Figures 5(f)–5(h)), and protein levels of p53 (Figure S2E) and p21 (Figure S2E). As we expected, administration of ISRIB could robustly deplete the inhibitory effect of CORM-A1 on downregulation of p53, p21, and SASP expression. Additionally, ISRIB also abrogated the inhibitory effect on the secretion of SASP (Figures 5(i)–5(l)).

Telomere shortening is an important marker of cellular senescence [1], and bleomycin has been reported to induce telomere shortening and cellular senescence to provoke the development of pulmonary fibrosis [35]. Moreover, several studies have proved that telomerase is a ribonucleoprotein that includes the telomerase reverse transcriptase (TERT) and the telomerase RNA (TERC) [36, 37]. Telomerase inhibition by targeting TERT has been shown to induce cellular senescence, shortening of telomere, and DNA damage [38]. Based on these studies, to verify whether exogenous CO could regulate telomere shortening via induction of SGs, we next checked the protein level of TERT (Figure S2E). Our results showed that bleomycin-mediated cellular senescence could interfere with the protein level of TERT, and CORM-A1 could significantly reverse the change of TERT. However, ISRIB cotreatment remarkably compromises the enhancement of TERT induced by CORM-A1. These results demonstrated that exogenous CO could upregulate TERT expression to control telomere shortening, and which might be associated with the formation of SGs, while the detailed molecular mechanisms should be clarified in further studies.

Taken together, our data clearly indicated that ISRIB could interfere with the antisenescent effect of CO on bleomycin-induced cellular senescence via diminishing SG assembly and sequestration of PAI-1 and influencing telomere shortening.

## 4. Discussion

Cellular senescence can induce both beneficial and deleterious outcomes in a context-dependent manner [4, 39], and these opposing effects mediated by senescent cells are strongly related to the upregulation of SASP, including growth factors, cytokines, and extracellular matrix metalloproteinase [40]. Accumulating evidences have supported that PAI-1, a member of SASP, is a critical marker of cellular senescence associated with aging and aging-related pathologies, such as metabolic syndrome, chronic kidney disease, and multiorgan fibrosis [7, 41]. One previous study has demonstrated that the formation of SGs induced by continuous mild stress can prevent replicative senescence by promoting the localization of PAI-1 into SGs, which can increase the nuclear translocation of cyclin D1 to delay the process of replicative senescence [28]. Recently, we have proved that CO possesses a beneficial effect on SG formation through the induction of ISR [29]. Based on these reports and our results, herein, we demonstrated for the first time that the assembly of SGs mediated by CO could sequestrate PAI-1, which protects cells against bleomycin-induced premature senescence.

In this study, we treated human diploid fibroblasts WI-38 cells and primary mouse embryonic fibroblasts with bleomycin to establish DNA damage-induced premature senescence. Bleomycin, one mixture of glycopeptide antitumor drug, is widely used for treatment of squamous cell carcinomas, testicular carcinoma, and Hodgkin's and non-Hodgkin's lymphomas [42]. Bleomycin is also one of the most toxic antineoplastic drugs for inducing lung fibrosis, due to epithelial cell injury with reactive hyperplasia, epithelial-mesenchymal transition, differentiation of fibroblasts to myofibroblasts, and the basement membrane and alveolar epithelium injuries [43]. Besides that, accumulating evidence suggested that bleomycin can also induce apoptosis and senescence in lung nonepithelial cells, such as lung fibroblasts, through oxidative stress-induced single- and double-stranded breaks in DNA [30]. To ameliorate these side effects on normal cells, here, we observed that the administration of exogenous CO revealed a strong antisenescent effect on bleomycin-mediated premature senescence. Our results showed that the administration of low-dose CORM-A1 and CO gas could significantly decrease multiple hallmarks of senescence, including the percentage of SA-*β*-gal-positive cells, DNA damage-associated *γ*-H2AX foci, cell cycle arrest-associated protein p53, and CDK inhibitor p21, as well as several SASPs (Figures 1 and 2 and S1 and S2). To validate the optimal concentration of CORM-A1, we first pretreated WI-38 cells with CORM-A1 in a dose-dependent manner and found that 40 *μ*M CORM-A1 showed most effectively to inhibit bleomycin-induced p21 and SASP (Figures 1(b)–1(e)). Moreover, cells treated with CORM-A1 for 6 h showed no cytotoxicity in WI-38 and MEF cells.

Given that PAI-1 is defined as a novel biomarker and a critical mediator of cellular senescence [7, 33, 41], we next evaluated whether PAI-1 was associated with bleomycin-induced lung fibroblast senescence. Consistent with the previous study that chemotherapeutic agent can induce the mRNA expression of PAI-1 in a glioblastoma cell strain [44], we also observed bleomycin-stimulated cells exerted a significant enhancement of PAI-1 in WI-38 cells (Figures 3(a) and S2D). Next, we silenced the expression of PAI-1 or used PAI-1 inhibitor to explain the crucial role of PAI-1 in bleomycin-induced premature senescence. Our data clearly suggested that, as cells transfected with siRNA against PAI-1 or cotreated with small molecular inhibitor, TM5441, the senescence-associated markers were all dramatically decreased. Consistent with the data reported in previous study [45], PAI-1 could regulate the p53 downstream signaling pathway, and PAI-1 gene silencing could significantly downregulate the protein levels of p53 and p21 to reduce the process of senescence (Figure S2D). These data firmly indicated again that PAI-1 is not only a mediator but also a therapeutic target for the bleomycin-induced lung fibroblast senescence.

In response to diverse environmental stresses, eukaryotic cells will activate defense mechanisms to control energy expenditure for the repair of stress-induced damage, and one of the important mechanisms to promote cell survival is induction of the SG assembly in the cytoplasm [27]. Recently, it has been reported that cellular senescence can interfere the formation of cytoplasmic SGs through the inhibition of SG-nucleating proteins [46]. However, continuous exposure to temperate oxidative stress can induce SG formation, which interferes the process of replicative senescence via sequestration of PAI-1 and then activates the nuclear translocation of cyclin D1 to attenuate cell cycle arrest [28]. CO has been proved as a novel inducer of SG formation via induction of the PERK-eIF2*α* signaling pathway, one of the three ISR branches [29]. Based on these reports, herein, we tried to evaluate the relationship between antisenescent effect of CO and its beneficial effect on SG formation (Figure 4). According to our results, in the challenge of bleomycin, CORM-A1 could induce the localization of PAI-1 into SGs through the aggregation of TIA-1 and PAI-1, which also dramatically reduced the secretion of PAI-1. In addition, our results revealed that CORM-A1 showed a mild inhibitory effect on PAI-1 transcription and that was not a main cause but partially interfere the secretion of PAI-1. Next, to point out the activity of PAI-1, we assessed its downstream signaling pathway and found that CORM-A1 could significantly induce the nuclear translocation of cyclin D1 (Figure 4(d)) and hyperphosphorylation of Rb (Figure 4(e)) in the stimulation of bleomycin. These results are consistent with previous study [28] that the increased formation of SGs by continuous mild stress could sequestrate PAI-1 and subsequently activates cyclin D1 nuclear translocation and delay the process of replicative senescence.

Integrated stress response (ISR) has been reported to trigger the formation of SGs by the phosphorylation of eIF2*α* and reduce the formation of 43S translation preinitiation complexes. However, ISR inhibitor (ISRIB) can block the phosphorylation of eIF2*α* and the formation of SGs [47]. To further assess whether ISRIB could abolish the antisenescent effect of CO by compromising SG assembly, we cotreated WI-38 cells with CORM-A1 and ISRIB followed by the challenge of bleomycin and found that ISRIB could significantly abrogate the antisenescent effect of CORM-A1 and recover the bleomycin-induced premature senescence (Figure 5). Moreover, with previous study [28], ISRIB could dramatically inhibit the sequestration of PAI-1 into CO-induced SGs, and the secretion of PAI-1 was reversely increased. All above data proved again that the formation of SGs enhanced by CORM-A1 played a pivotal role in the prevention of bleomycin-induced cellular senescence rather than the control of PAI-1 transcripts and suggested that ISR is important for CO-mediated antisenescent function. Herein, we also observed that the administration of bleomycin could decrease the protein level of TERT, one critical component of telomerase [36, 37], and which might be associated with telomere shortening during the process of cellular senescence. Interestingly, as cells were pretreated with CORM-A1, the level of TERT showed a significant elevation, while the combinatorial treatment of ISRIB drastically abrogated the beneficial effect of CORM-A1 on TERT expression, suggesting that CO could regulate bleomycin-induced telomere shortening through upregulation of TERT, and which might be related to ISR activation and the SG formation. For the further study, we planned to determine that how CO enhances TERT and what role of CO-induced SGs plays in TERT expression and telomere shortening.

**Figure 6:**
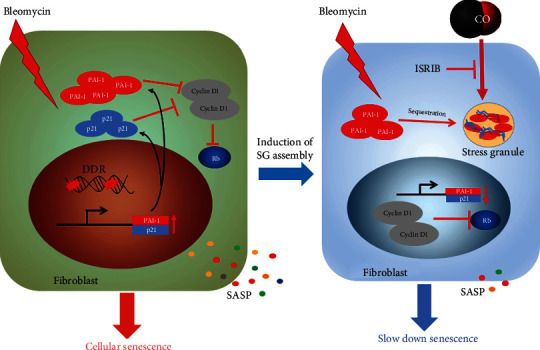
The molecular mechanism responsible for CO-mediated SG formation attenuates bleomycin-induced cellular senescence.

In conclusion, our work strongly demonstrated that the gasotransmitter CO could protect lung fibroblasts against bleomycin-induced premature senescence by inducing SG formation, promoting the sequestration of PAI-1 into SGs, as well as controlling the telomere shortening. These effects could activate cyclin D1 and hyperphosphorylation of Rb to delay the process of cellular senescence (Figure 6). We propose that the utilization of gaseous transmitter, CO, can be a novel therapeutic strategy to prevent bleomycin-mediated cellular senescence and its side effects associated with pathogenesis.

## Data Availability

All data needed to evaluate the conclusions in the paper are present in the paper and/or Supplementary Materials. Additional data related to this paper may be requested from the authors.

## Abbreviations

BLM: Bleomycin
CDK1: Cyclin-dependent kinase 1
CO: Carbon monoxide
CORM-A1: CO-releasing molecular-A1
DDR: DNA damage response
eIF2*α*: Eukaryotic translation initiation factor 2A
FGF21: Fibroblast growth factor 21
G3BP1: GTPase-activating protein-binding protein 1
*γ*-H2AX: Phosphorylated H2A histone family member X
HO: Heme oxygenase
H_2_S: Hydrogen sulfide
IL-6: Interleukin-6
IL-1*β*: Interleukin-1 beta
ISR: Integrated stress response
ISRIB: ISR inhibitor
mTOR: Target of rapamycin
mtROS: Mitochondrial reactive oxygen species
NF-*κ*B: Nuclear factor-kappa B
NO: Nitric oxide
PAI-1: Plasminogen activator inhibitor
PERK: Protein kinase R-like endoplasmic reticulum kinase
SA-*β*-gal: Senescence-associated *β*-galactosidase
SASP: Senescence-associated secretory phenotype
SESN2: Upregulated sestrin 2
SG: Stress granules
TERT: Telomerase reverse transcriptase
Tg: Thapsigargin
TIA-1: T cell intracytoplasmic antigen
TNF-*α*: Tumor necrosis factor alpha.

## Supplementary Materials

Supplementary Materials Figure S1: low-dose CO gas ameliorates BLM-induced cellular senescence in WI-38 cells. (A) WI-38 cells were treated with CO gas (250 ppm) in a time-dependent manner (0, 6, 12, and 24 h), and MTT assay was performed to detect cell viability. WI-38 cells were pretreated with CO gas (250 ppm) for 6 h followed by the challenge of BLM (25 *μ*g/ml) for 48 h. Then, SA-*β*-gal staining (B) and *γ*-H2AX foci (C) were detected, and the mRNA expressions of p21 (D), IL-6 (E), TNF-*α* (F), and IL-1*β* (G) were detected by qRT-PCR. Quantitative data are expressed as the means ± SD (*n* = 3 determined in three independent experiments). ^∗^p < 0.05, ^∗∗^*p* < 0.01, and ^∗∗∗^*p* < 0.001. Figure S2: protein levels of p53, p21, PAI-1, and TERT in different groups. (A–C) WI-38 cells were pretreated with CORM-A1 (40 *μ*M) for 6 h followed by the stimulation of BLM (25 *μ*g/ml) for 96 h. During the process of senescence, cells were posttreated with CORM-A1 (40 *μ*M) for 6 h every other day. After a 4-day incubation, the protein levels of p53 and p21 were measured by Western blot assay. (D) WI-38 cells were transfected with scramble siRNA (scRNA) and siRNA against PAI-1 (siPAI-1) for 36 h and then treated with BLM (25 *μ*g/ml) for 96 h. Protein levels of p53, p21, and PAI-1 were detected by Western blot assay. (E–H) WI-38 cells were pretreated with CORM-A1 (40 *μ*M) with or without ISRIB (200 nM) for 6 h followed by the challenge of bleomycin (25 *μ*g/ml) for 96 h. Then, protein levels of p53, p21, and TERT were assessed by Western blot. Bar graphs are summary data of normalized densitometric ratios. Quantitative data are expressed as the means ± SD (*n* = 3 determined in three independent experiments). ^∗^*p* < 0.05 and ^∗∗∗^*p* < 0.001.
