# Supplementary material for: Gasotransmitter CO Attenuates Bleomycin-Induced Fibroblast Senescence via Induction of Stress Granule Formation
Source: Oxid Med Cell Longev. 2021 Jun 29;2021:9926284. doi: 10.1155/2021/9926284 (PMC8263219; doi:10.1155/2021/9926284)
Supplement: Supplementary Materials — Figure S1: low-dose CO gas ameliorates BLM-induced cellular senescence in WI-38 cells. (A) WI-38 cells were treated with CO gas (250 ppm) in a time-dependent manner (0, 6, 12, and 24 h), and MTT assay was performed to detect cell viability. WI-38 cells were pretreated with CO gas (250 ppm) for 6 h followed by the challenge of BLM (25 μg/ml) for 48 h. Then, SA-β-gal staining (B) and γ-H2AX foci (C) were detected, and the mRNA expressions of p21 (D), IL-6 (E), TNF-α (F), and IL-1β (G) were detected by qRT-PCR. Quantitative data are expressed as the means ± SD (n = 3 determined in three independent experiments). ∗p < 0.05, ∗∗p < 0.01, and ∗∗∗p < 0.001. Figure S2: protein levels of p53, p21, PAI-1, and TERT in different groups. (A–C) WI-38 cells were pretreated with CORM-A1 (40 μM) for 6 h followed by the stimulation of BLM (25 μg/ml) for 96 h. During the process of senescence, cells were posttreated with CORM-A1 (40 μM) for 6 h every other day. After a 4-day incubation, the protein levels of p53 and p21 were measured by Western blot assay. (D) WI-38 cells were transfected with scramble siRNA (scRNA) and siRNA against PAI-1 (siPAI-1) for 36 h and then treated with BLM (25 μg/ml) for 96 h. Protein levels of p53, p21, and PAI-1 were detected by Western blot assay. (E–H) WI-38 cells were pretreated with CORM-A1 (40 μM) with or without ISRIB (200 nM) for 6 h followed by the challenge of bleomycin (25 μg/ml) for 96 h. Then, protein levels of p53, p21, and TERT were assessed by Western blot. Bar graphs are summary data of normalized densitometric ratios. Quantitative data are expressed as the means ± SD (n = 3 determined in three independent experiments). ∗p < 0.05 and ∗∗∗p < 0.001. [file 9926284.f1.docx]

**Supplemental Files**

**Gasotransmitter CO attenuates bleomycin-induced fibroblast senescence via induction of stress granule formation**

Yingqing Chen,^1^ Feng Jiang,^1^ Guangyao Kong,^1^ Shuo Yuan,^2^ Yuying Cao,^1^ Qinggao Zhang, ^1,2*^ Qianqian Wang,^1*^and Liping Liu,^1*^

^1^ *Chronic Disease Research Center, Medical College, Dalian University, Dalian 116622, Liaoning, China.*

^2^ *Department of Immunology and Pathogenic Biology, Yanbian University College of Basic Medicine, Yanji, 133022, Jilin, China.*

*** Address correspondence to: Qinggao Zhang, [zhangqinggao@dlu.edu.cn](mailto:zhangqinggao@dlu.edu.cn); Qian qian Wang, [wangqianqian@dlu.edu.cn;](mailto:wangqianqian@dlu.edu.cn;) Liping Liu, [1106548638@qq.com](mailto:1106548638@qq.com;)**


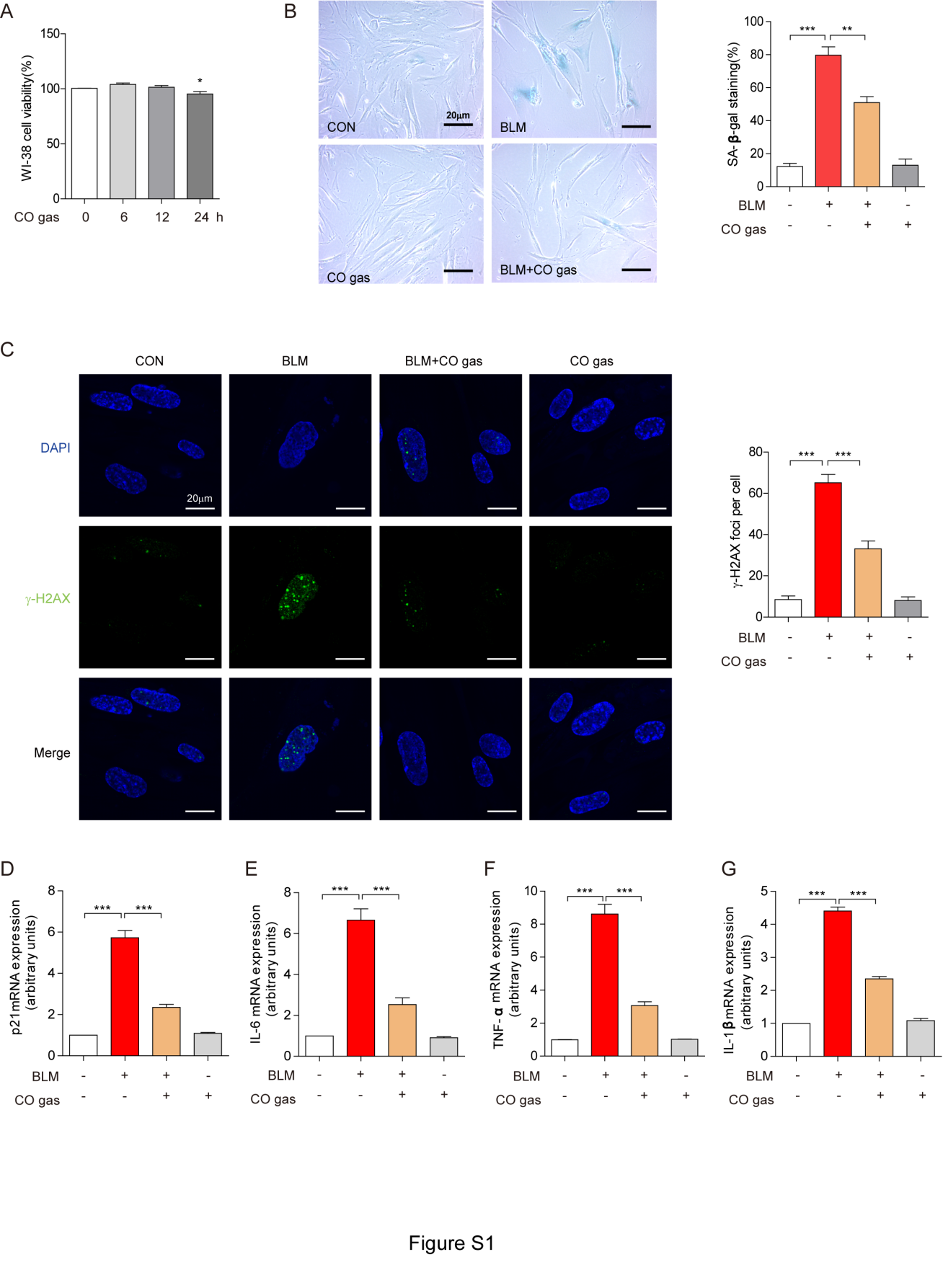


**Figure S1. Low dose CO gas ameliorates BLM-induced cellular senescence in WI-38 cells.** (A) WI-38 cells were treated with CO gas (250ppm) in time dependent manner (0, 6, 12 and 24h), and MTT assay was performed to detect cell viability. WI-38 cells were pre-treated with CO gas (250ppm) for 6h followed by the challenge of BLM (25μg/ml) for 96h. Then SA-β-gal staining (B) and γ-H2AX foci (C) were detected, and the mRNA expression of p21(D), IL-6(E),TNF-α(F), and IL-1β(G) were detected by qRT-PCR.Quantitative data are expressed as means±SD (n=3 determined in three independent experiments). ^*^*p*<0.05, ^**^*p<*0.01, and ^***^*p<*0.001.


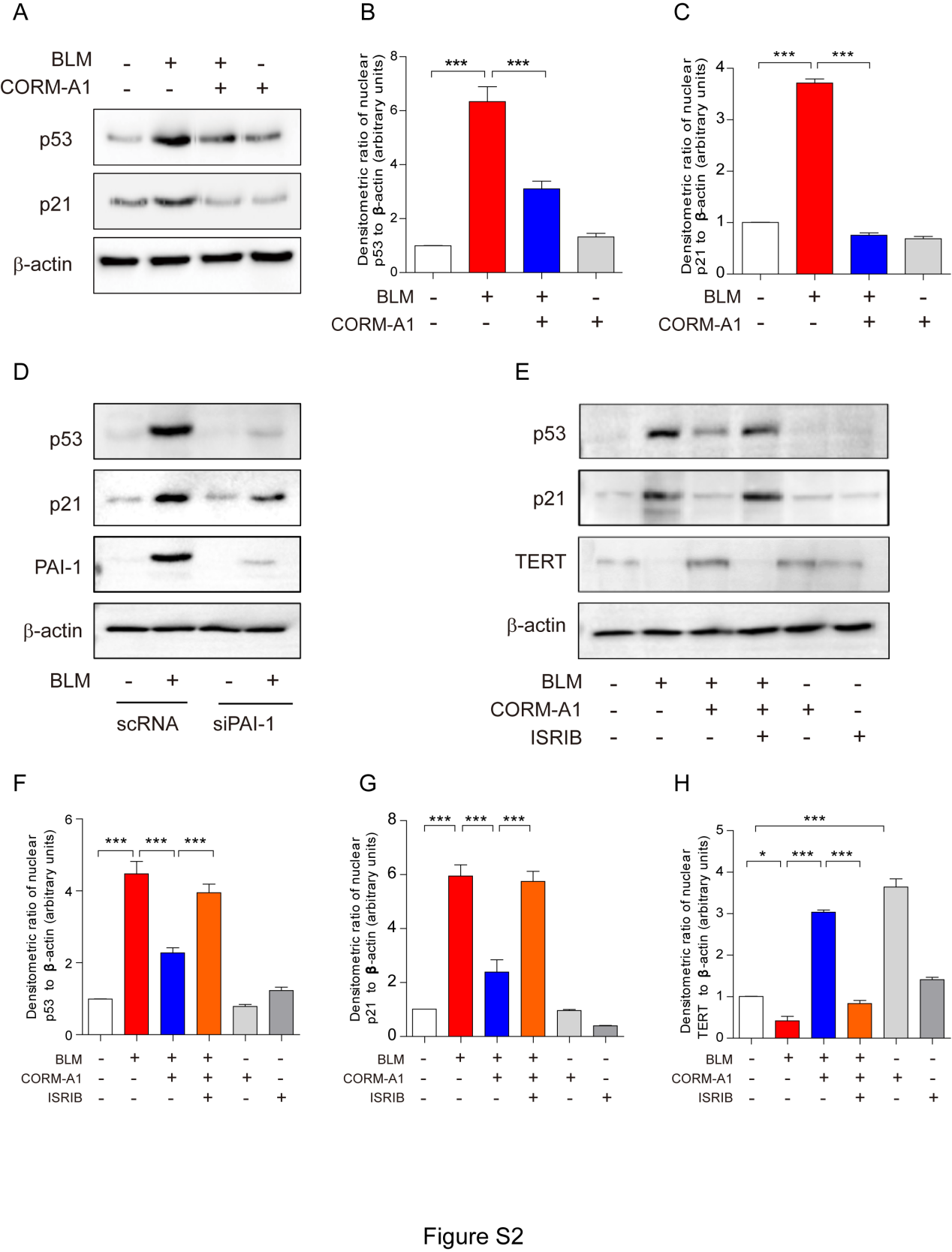


**Figure S2. Protein levels of p53，p21，PAI-1 and TERT in different groups.** (A-C) WI-38 cells were pre-treated with CORM-A1 (40μM) for 6h followed by the stimulation of BLM (25μg/ml) for 96h. During the process of senescence, cells were post-treated with CORM-A1(40μM) for 6h every other day. After 4-day incubation, the protein levels of p53 and p21 were measured by Western blot assay. (D) WI-38 cells were transfected with scramble siRNA (scRNA) and siRNA against PAI-1 (siPAI-1) for 36 h, and then treated with BLM (25μg/ml) for 96h. Protein levels of p53, p21 and PAI-1 were detected by Western blot assay. (E-H) WI-38 cells were pre-treated with CORM-A1 (40 μM) with or without ISRIB (200 nM) for 6h followed by the challenge of bleomycin (25μg/ml) for 96h. Then protein levels of p53, p21 and TERT were assessed by Western blot. Bar graphs are summary data of normalized densitometric ratios. Quantitative data are expressed as means±SD (n=3 determined in three independent experiments). ^*^*p*<0.05 and ^***^*p<*0.001.
